# Helmet use in equestrian athletes: opportunities for intervention

**DOI:** 10.2217/cnc-2020-0019

**Published:** 2020-12-14

**Authors:** Ansley Grimes Stanfill, Kayla Wynja, Xueyuan Cao, Drew Prescott, Sarah Shore, Brandon Baughman, Anthony Oddo, Jack W Tsao

**Affiliations:** 1 Department of Acute & Tertiary Care, University of Tennessee Health Science Center, 920 Madison Ave., Memphis, TN 38163, USA; 2 Department of Neurology, University of Tennessee Health Science Center, 920 Madison Ave., Memphis, TN 38163, USA; 3 Semmes Murphey Clinic, 6325 Humphreys Blvd, Memphis, TN 38120, USA; 4 Department of Neurosurgery, University of Tennessee Health Science Center, 920 Madison Ave., Memphis, TN 38163, USA; 5 Department of Pediatrics, University of Tennessee Health Science Center, 920 Madison Ave., Memphis, TN 38163, USA; 6 Children’s Foundation Research Institute, Le Bonheur Children’s Hospital, 50 N. Dunlap St, Memphis, TN 38163, USA

**Keywords:** concussion, horse riding, traumatic brain injury

## Abstract

**Background:**

Equestrian athletes (horse riders) are at high risk for head injury, including concussions.

**Materials & methods:**

Adults riders were recruited via social media posting to complete a branching survey collecting data on demographics, riding experience, helmet use, injury history and concussion symptom knowledge. Results are reported as frequencies and percentages, with associations tested using chi-square with significance level p < 0.05.

**Results:**

Of the 2598 subjects, about 75% reported always wearing a helmet. Of those who did not, the most common reasons were that helmets are unnecessary (57.4%) or do not fit well (48.6%). Many indicated improper storage conditions and/or did not follow manufacturer’s replacement recommendations. Most (75.4%) reported a high level of comfort with recognizing concussion signs, with half experiencing a prior head injury.

**Conclusion:**

This information suggests opportunities for intervention to improve helmet use through increased fit, while the responses indicate a need for further education on proper helmet use.

Despite the high risk of head injury in equestrian activities, helmets are still under-utilized, with as few as 9% of adult riders wearing helmets on a regular basis [1–3]. The rate of serious horseback riding injuries per number of riding hours is estimated to be higher than that of motorcyclists and automobile racers [1,2], and these injuries account for approximately 103,000 emergency department admissions each year [4]. Head injuries, such as concussions and other more severe traumatic brain injuries, are common during equestrian sports and account for approximately 20% of riding-related injuries [3,4]. Given that nearly 40 million Americans are involved in equestrian competitions and many more participate in recreational equestrian activities, lack of helmet use is a serious public health safety concern [3,5,6].

Modern American Society for Testing and Materials/Safety Equipment Institute-approved helmets have reduced riding-related head injuries by 30% and severe head injuries by 50%, leading national competitive organizations to publicly acknowledge the importance of proper helmet use [7]. In particular, the US Pony Club and the US Equestrian Foundation (both KY, USA) have published guidelines for the use of a certified helmet for riders involved in equestrian competitions [4,7]. Still, there is a need for further regulation, as there are currently no laws in any US state regarding helmet use during noncompetitive events, private training or casual riding and many riders still do not wear a helmet on a regular basis. For those equestrians who do use a helmet, it has been observed by the authors that many may fail to maintain this vital piece of equipment carefully.

Despite these concerning descriptors, little research has been conducted to identify key factors related to the use or nonuse of helmets, helmet handling and riders’ response to head injury, especially concussion, during horse-related activities. There is also a lack of clarity concerning equestrians’ level of awareness and knowledge of concussion symptoms should a head injury occur. The purpose of this study is to identify the reasons helmets are improperly worn, cared for or not used and to survey equestrians’ knowledge of the common symptoms of head injury.

## Materials & methods

### Survey development

This research was developed with the support of the local equestrian community of the lead author. This informal group comprised both professional and amateur participants in a broad variety of equestrian disciplines. These individuals did not have specialized medical or research training but were interested in the overarching purpose of this project due to their equestrian activities. The lead author initially identified the research questions and priorities for this survey in conjunction with the local equestrians’ experiences and preferences. During the development process, potential questions were shared with these individuals and the authors worked iteratively with this informal group to ensure their ideas were captured and to maintain content validity of the final survey.

### Participants

Following Institutional Review Board approval, the final online branching survey was made available to the public through the official Facebook and Twitter pages of the college so that it could be ‘shared’ by individual accounts of interested parties. In the post, individuals (both male and female) over the age of 18 years who regularly ride horses at least once a month were asked to complete the survey by clicking on an embedded and anonymous link. Once potential participants opened the link, they were advised via an informed consent notice that no personally identifiable information would be collected through the survey nor were any data recorded related to the electronic location of the survey completion. Potential participants were also informed that participation was completely optional, the survey could be discontinued at any time and the contact information of the Principal Investigator and Institutional Review Board chairman was provided should the participant have any further questions or concerns. By clicking ‘continue’ at the bottom of the webpage, participants confirmed that they were providing informed consent to begin the survey.

### Survey instrument

**Figure 1.**
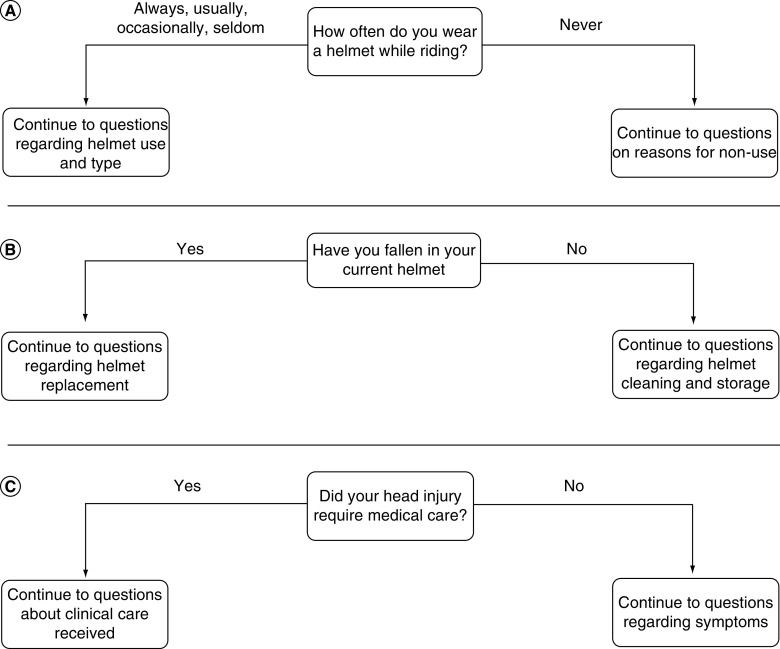
Illustrations of three key branching points of the survey (A–C).

A sample illustration to demonstrate the branching pattern for survey questions is provided in Figure 1. Data were collected in the following categories: age; riding experience; helmet use, price and storage and care conditions; length of time the helmet has been owned; circumstances under which a helmet is usually replaced; reasons why a helmet is not worn; and circumstances that would improve helmet use. In order to better understand equestrians’ knowledge of concussion and treatment of head injury, participants were surveyed about previous head injury, symptoms and general descriptions of medical care received after such events occurred. The respondents were also questioned about possible signs of a head injury and how such events would be handled if the individual were suspicious of their own injury or suspicious of a riding companion’s injury.

### Statistics

Descriptive statistics on the responses to each data category were calculated using R statistical software [8] and presented as frequencies and percentages. The association of helmet usage or nonusage with demographic variables was tested in Chi-squared tests with p-values determined by Monte Carlo simulations. Significance level was set at p < 0.05 without multiple testing adjustment.

## Results

The social media link to the survey was active for 30 days. In the first week, the original social media post generated 120 comments, was re-shared more than 650-times and generated 2611 anonymous responses from a broad demographic sample of participants. From these participants, 13 did not provide one or more answers to the four key demographic questions: “How old are you?”; “How many years of riding experience do you have?”; “Are you an amateur or professional rider?”; and/or “How often do you ride?”. These participants were therefore excluded from further analysis as the authorship team felt that nonanswers to these first key questions may have been indicative of technical difficulties with the survey. Thus, a total of 2598 participants were included in the remaining analyses.

Our respondents were fairly well distributed across age brackets, though most were middle-aged adults (Table 1). The majority of the participants (55.4%; n = 1440/2598) identified as having 20+ years of experience and were amateur riders (85.5%; n = 2221/2598). About 47.7% (n = 1240/2598) of participants reported riding a few days a week, with 26.4% riding competitively and 44.4% participating in both competitive and leisure riding activities.

**Table 1.** Demographics of survey respondents (n = 2598).

Question	n (%)	How often do you wear a helmet while riding?					p-value
		Always (n = 1943) n (%)	Usually (n = 282) n (%)	Occasionally (n = 116) n (%)	Seldom (n = 107) n (%)	Never (n = 150) n (%)	
How old are you?							
18–25	531 (20.4)	342 (64.4)	79 (14.9)	38 (7.2)	26 (4.9)	46 (8.7)	<0.001
25–35	588 (22.6)	396 (67.4)	84 (14.3)	34 (5.8)	33 (5.6)	41 (7.0)	
35–55	807 (31.1)	640 (79.3)	75 (9.3)	23 (2.9)	26 (3.2)	43 (5.3)	
55–65	469 (18.1)	386 (82.8)	33 (7.1)	15 (3.2)	16 (3.4)	16 (3.4)	
65+	203 (7.8)	176 (86.7)	11 (5.4)	6 (3.0)	6 (3.0)	4 (2.0)	
How many years of riding experience do you have?							
0–5	120 (4.6)	93 (77.5)	18 (15.0)	3 (2.5)	3 (2.5)	3 (2.5)	0.133
5–10	201 (7.7)	157 (78.1)	20 (10.0)	9 (4.5)	6 (3.0)	9 (4.5)	
10–15	424 (16.3)	311 (73.4)	48 (11.3)	24 (5.7)	12 (2.8)	29 (6.8)	
15–20	413 (15.9)	285 (69.0)	54 (13.1)	20 (4.8)	23 (5.6)	31 (7.5)	
20+	1440 (55.4)	1094 (76.1)	142 (9.9)	60 (4.2)	63 (4.4)	78 (5.4)	
Are you an amateur or professional rider?							
Amateur	2221 (85.5)	1686 (76.0)	231 (10.4)	93 (4.2)	91 (4.1)	117 (5.28)	0.004
Professional	377 (14.5)	254 (67.4)	51 (13.5)	23 (6.1)	16 (4.2)	33 (8.8)	
How often do you ride?							
Daily	561 (21.6)	422 (75.2)	65 (11.6)	21 (3.7)	22 (3.9)	31 (5.5)	0.089
A few days a week	1240 (47.7)	940 (76.0)	130 (10.5)	61 (4.9)	44 (3.6)	62 (5.0)	
At least once a week	531 (20.4)	384 (72.3)	60 (11.3)	20 (3.8)	34 (6.4)	33 (6.2)	
At least once a month	266 (10.2)	194 (72.9)	27 (10.2)	14 (5.26%)	7 (2.6)	24 (9.1)	

The vast majority of respondents reported that they always wore a helmet (75.8%; n = 1969/2598). When considering only professionals, 67.4% (n = 254/377) reported that they always wore a helmet. However, of the respondents that usually, sometimes, seldom or never wore a helmet (n = 629/2598), 48.6% (n = 306/629) selected that they would wear a helmet, if it had a better fit. Of those who did not always wear a helmet, their reasoning for sporadic use was that they did not want to (41.4%; n = 260/629), they forgot (36.3%; n = 228/629), the helmet was too hot (44.3%; n = 279/629) or the helmet was generally uncomfortable (37.5%; n = 236/629). Of these respondents, 57.4% (n = 361/629) also selected that they believe helmet use is unnecessary. A total of 15.6% (n = 98/629) selected that they would not wear a helmet more frequently under any circumstances. The frequency of helmet use was significantly associated with age (p < 0.001) and professional status (p = 0.004) for our sample.

Of our 2598 respondents, 2434 responded to questions on helmet ownership and care conditions. The majority of those that owned a helmet (39.3%; n = 957/2434) reported that their helmet cost between $50–100 USD, with many having owned their helmet for less than 5 years (92.5%; n = 2251/2434) and planning to replace it within a 5-year time frame (56.8%; n = 1383/2434). Most (83.4%; n = 2030/2434) stored their helmet either in a car or in a barn. In total, 28.2% (n = 686/2434) reported having fallen in their current helmet, and only 4.8% (n = 33/686) followed the recommendations of most manufacturers to replace the helmet after a fall.

Out of the 2492 (95.9%; n = 2492/2598) participants that answered the question “Have you ever received a head injury while riding?”, 1325 of these (53.2%) responded that they had received such injury while 149 (6%) reported that they were unsure. Of note, the survey did not provide a definition of head injury, and the term was open to interpretation by the respondent. Of those that did report an injury, 67.5% (n = 894/1325) reported getting back on their horse immediately following injury. About 42% of those who were injured did not seek medical care (n = 562/1325), while the remainder (n = 763/1325) did receive some medical care, with the most common symptoms selected for these individuals being headache (n = 667/763; 87.4%), dizziness (n = 510/763; 66.8%) and feeling “off” (n = 398/763; 52.2%). For those that received medical care for their symptoms, 77% (n = 588/763) went to the emergency department but were not admitted and 14% (n = 107/763) were hospitalized. Fortunately, only 2% (n = 15/763) of our respondents required surgery, and only 6% (n = 46/763) spent time in an intensive care unit. Still, in response to our question “If you fell alone, what would you do?”, many respondents indicated that they would seek some form of medical or nonmedical assistance, but 17.7% (n = 328/1855) of respondents to this question noted they would get back on the horse immediately while 4.8% (n = 74/1855) would not have access to help from others.

The majority of our respondents felt either “very comfortable” or “fairly comfortable” with determining the signs and symptoms of concussion (n = 1666/2210; 75.4%). Those that reported feeling “very comfortable” could correctly identify most of the signs of concussion, but a minority still missed some common symptoms of concussion (n = 775; Table 2).

**Table 2.** Percentage of respondents who felt “very comfortable” recognizing concussive symptoms (n = 775) and also recognized the following common symptoms of head injury.

Symptom	Correctly identified the symptom (%)
Headache	98.6
Dizziness	98.7
Balance issues	94.7
Drowsiness	94.2
Memory problems	97.0
Loss of consciousness	77.98
Nausea or vomiting	97.8

## Discussion

Although a high rate of equestrian activity-related head injuries continues to occur, there concurrently remains a lack of information about how equestrians decide to use or not use helmets, how they care for this important piece of equipment and how they identify and seek treatment for head injury if it occurs. This study addresses all of these concerns and, due to propagation through social media, provides respondent data from a wide variety of demographic characteristics, suggesting broad applicability of the findings.

Approximately 15% of the respondents that did not ever wear a helmet during equestrian activities indicated that they would not wear a helmet under any conditions, while 75% report that they always wear a helmet. This is a slightly higher rate than some other helmeted sports such as snow sports (70% [9]) and cycling (50% [10]). Still, our results provide several opportunities for intervention for those that either could be persuaded or those that sometimes (i.e., those who provided responses in the usually, occasionally and seldom categories) wore a helmet. Importantly, our results indicate that equestrians are overwhelmingly desirous of better fitting, more comfortable and better ventilated helmets. This was especially true for our leisure riders (noncompetitive) as this demographic selected comfort above all other possible reasons for not always wearing a helmet. Despite the increasing cost of some more fashionable helmet models (up to $600+ USD), many American Society for Testing and Materials/Safety Equipment Institute-certified helmets are in the $50–100 USD range. Expense was not found to be a major factor overall in helmet nonuse by demographic characteristics, although there were significant differences in this response for those in our youngest demographic brackets.

Our results also demonstrate that to better protect equestrians, development and improvement of helmet quality and comfort may be necessary [11]. Our respondents that did not always wear a helmet indicated that the number one reason was comfort and fit, so improvement in these areas may increase helmet use. Write-in comments on the survey and social media postings further indicated an awareness that head injuries may still occur even with helmet use, so this may highlight an educational opportunity for manufacturers to better advertise safety standards and the limitations of the level of protection offered. A similar study to ours found that there was low awareness among equestrians of the inability of helmets to protect against concussion [12]. While helmets can greatly improve outcomes after falls [13], current safety inspections and helmet-testing occur through impact with hard, flat surfaces as opposed to ‘real-world’ injuries that often occur with a rotational component to the fall [14]. The recent advancements in helmet technology (such as the MIPS system) are promising in laboratory testing, which may help to protect riders more completely in such falls and could be better marketed to demonstrate the advancements in injury protection they may offer for riders [15].

Despite these advancements, even the best technology does not work if helmets are not worn, are stored in improper conditions or are not replaced after a potentially damaging blow. Our results also indicate that equestrians need better education to know how to store, clean and replace helmets frequently, and are in agreement with other work in this area [12]. Those riders who do wear helmets on a regular basis to protect themselves but do not follow helmet care guidelines leave themselves more vulnerable to injury than they may perceive. Helmets should be stored at room temperature, out of direct sunlight and properly cleaned on a regular basis to prevent helmet breakdown and ensure adequate protection. Helmets that have been stored in direct sunlight, stored in hot temperatures or left dirty with makeup, sweat and sunscreen can experience a breakdown of the interior lining, potentially leading to ineffective protection in the event of a fall [16]. Furthermore, helmets should also be replaced following any fall, any blow to the head while wearing the helmet (e.g., contact with a tree branch on a trail ride) or if they are dropped. Most manufacturers’ websites (such as Troxel, Charles Owen and others) recommend replacing helmets after 5 years of use even if a fall or blow has not occurred, as the materials degrade even under the best conditions. The majority of our respondents stored their helmets either in a car or in a barn, two locations where the helmet may be exposed to high temperatures, and therefore, be prone to breakdown. Over 40% of respondents had no plans to replace their current helmet within the manufacturer-recommended 5-year time frame, and of those that had fallen, only 4.8% replaced the helmet they wore after the fall.

Our results are also concerning, as they highlight an issue of the equestrian culture and one in which there is opportunity for intervention to increase safety. Many famous trainers have advocated for always getting back on the horse after a fall; this advice stems from the idea that riders should remount to avoid teaching horses to throw riders off to ‘get out of work’. While it is possible that horses may learn such behavior, the adage has become threaded into equestrian culture so deeply that unless injury is obvious (i.e., blood or displaced limbs), peer pressure to remount may supersede any concerns of a less obvious but still serious injury, such as concussion. Indeed, the invisibility of a head injury [17] and peer pressure to perform are common risk factors that can lead to concussion underreporting behaviors in many sports [18]. A too-early return to activities exacerbates symptoms, raises the risk for long-term sequelae [19,20] and can place riders at risk for further injury due to neurological changes that occur in concussion (i.e., balance problems, dizziness). Fortunately, the majority of our respondents correctly identified many of the more common concussion symptoms. Our results are in agreement with a similar study done among New Zealand equestrians [12] with similar rates being reported in motocross participants [21] and soccer players [22].

It is a limitation of this study that participants were not queried about less common concussion symptoms, outside of those listed in Table 2. Regardless, the subjectivity of concussion symptoms, the variability of symptom presentation [23], and reliance from self-report of the injured person make it imperative that education is provided to begin to change riding culture and reduce the stigma of seeking medical attention.

In order to rapidly reach a large number of equestrian athletes in a wide variety of ages, disciplines and experience levels, this broad-stroke survey was launched using social media. The ease and anonymity of reporting was a major advantage, but there were also drawbacks. Due to an initial flood of responses as the survey opened, some technical issues were encountered with the university’s server. Some of the initial participants were not able to type input under the “other” option provided for some questions. Others (especially those using older mobile devices) found that the survey would crash or would not open altogether. These issues were quickly corrected by the university’s information technology department, allowing the majority of participants to fully answer the survey but limiting the sample size for some specific questions.

After completion of the survey, many participants returned to the original social media post to tell their personal riding and helmet stories. This allowed for a vibrant, but unregulated, conversation between riders which may have introduced some bias if seen by new survey respondents. Still, the overarching theme of these communications was the desire for respondents to have their individual voice and story heard, a desire which cannot be met through the anonymous survey method discussed here. Although this survey still provided valuable information for possible intervention, these communications highlight the need for a qualitative approach that should be used in future research, as this approach can more accurately capture the passion equestrians have in discussing this topic.

## Conclusion

This study used social media to survey helmet use in equestrian riding and determine the general knowledge of concussion symptoms and management. Although recent efforts by the US Equestrian Federation, among others, have focused on promoting helmet use and increasing rider awareness regarding signs of concussion following an equestrian injury [1,4], such information must also be expanded further; our results indicated that many equestrians are improperly caring for their helmets and may continue to ride after an injury is sustained. The results from this survey will be used to develop equestrian-specific interventions to increase proper helmet use, which will in turn aim to reduce the number and severity of head injuries acquired during equestrian sports.

## Future perspective

In the coming years, advancements in comfort and technology will continue to improve the protection offered by helmets. Future research should include a qualitative component but should focus on educational interventions to improve knowledge of proper helmet safety and better identification and care of concussion symptoms among equestrian athletes.

**Summary points**

- Helmets are underutilized in equestrian riding activities and while guidelines have been developed for some competitive activities, change has been slow to reach all types of equestrian activities and riding culture discourages riders from seeking medical attention following a fall or head injury.
- This is problematic, given the incidence of concussion in equestrian injury is double that of many other high-impact sports, including boxing, soccer, rugby and football.
- The results from this study demonstrate a need for efforts to increase riders’ awareness of head injuries and highlight the need for increased education for equestrians on helmet use, care and replacement.
